# Long-Reads Reveal That the Chloroplast Genome Exists in Two Distinct Versions in Most Plants

**DOI:** 10.1093/gbe/evz256

**Published:** 2019-11-21

**Authors:** Weiwen Wang, Robert Lanfear

**Affiliations:** Division of Ecology and Evolution, Research School of Biology, Australian National University, Acton, Australian Capital Territory, Australia

**Keywords:** single copy inversion, flip-flop recombination, chloroplast genome structural heteroplasmy

## Abstract

The chloroplast genome usually has a quadripartite structure consisting of a large single copy region and a small single copy region separated by two long inverted repeats. It has been known for some time that a single cell may contain at least two structural haplotypes of this structure, which differ in the relative orientation of the single copy regions. However, the methods required to detect and measure the abundance of the structural haplotypes are labor-intensive, and this phenomenon remains understudied. Here, we develop a new method, Cp-hap, to detect all possible structural haplotypes of chloroplast genomes of quadripartite structure using long-read sequencing data. We use this method to conduct a systematic analysis and quantification of chloroplast structural haplotypes in 61 land plant species across 19 orders of Angiosperms, Gymnosperms, and Pteridophytes. Our results show that there are two chloroplast structural haplotypes which occur with equal frequency in most land plant individuals. Nevertheless, species whose chloroplast genomes lack inverted repeats or have short inverted repeats have just a single structural haplotype. We also show that the relative abundance of the two structural haplotypes remains constant across multiple samples from a single individual plant, suggesting that the process which maintains equal frequency of the two haplotypes operates rapidly, consistent with the hypothesis that flip-flop recombination mediates chloroplast structural heteroplasmy. Our results suggest that previous claims of differences in chloroplast genome structure between species may need to be revisited.

## Introduction

Chloroplasts are organelles which are vital for photosynthesis. Most land plant chloroplast genomes are 120–160 kb in size (Wicke et al. 2011; Zheng et al. 2017), and have a quadripartite structure consisting of a pair of identical rRNA-containing inverted repeats (hereafter referred to as IR) of ∼10–30 kb divided by a large single copy (LSC) region of ∼80–90 kb and a small single copy (SSC) region of ∼10–20 kb.

Surprisingly, chloroplast genomes can exist two structural haplotypes differing in the orientation of single copy regions (Palmer 1983). The presence of this structural heteroplasmy has been confirmed in some land plants (Palmer 1983; Brears et al. 1986; Stein et al. 1986; Martin et al. 2013) and algae (Bohnert and Löffelhardt 1982; Aldrich et al. 1985), but its cause remains unknown. One hypothesis to explain the presence of structural heteroplasmy is known as flip-flop recombination (Stein et al. 1986). This hypothesis suggests that the large IRs could mediate frequent intramolecular recombination, resulting in the maintenance of roughly equal amounts of the two haplotypes differing only in the orientation of their single copy regions.

A better understanding of chloroplast genome structural heteroplasmy is important for a number of reasons. First, some recent papers have suggested that the orientation of the single copy regions differ between species (Ibrahim et al. 2006; Yang et al. 2010; Liu et al. 2013; Zhang et al. 2014; Wang et al. 2015), but Walker et al. (2015) pointed out that these studies seem to have overlooked the possibility that both orientations may coexist in a single individual. Second, the relationship between the structure of the chloroplast genome and the existence or otherwise of structural heteroplasmy remains poorly understood. For example, if flip-flop recombination causes chloroplast structural heteroplasmy, then it seems likely that the presence of two long IRs may be a prerequisite for the existence of heteroplasmy. Consistent with this, no heteroplasmy was observed in a chloroplast genome with highly reduced IRs (Wu, Lin, et al. 2011), but the generality of this observation remains to be tested. Third, if heteroplasmy is the norm rather than the exception, then this may represent a challenge for the assembly of chloroplast genomes, which may be easily overcome by simply allowing for the existence of two structural haplotypes during the assembly process. A large-scale analysis across many different species has the potential to provide a more complete picture of chloroplast heteroplasmy, further elucidating this fascinating phenomenon and potentially improving genome assembly and inference. In this study, we perform this large-scale analysis using a new method which we developed to quickly and conveniently quantify structural heteroplasmy in chloroplast genomes from long-read sequencing data.

Currently, there are two methods to detect different structural heteroplasmy in chloroplast genomes: Bacterial Artificial Chromosomes (BAC)-End-Sequence (BES) (Martin et al. 2013), and restriction digests (Stein et al. 1986). For the BES method, BAC libraries are constructed in which large pieces of whole genome DNA are inserted into bacterial chromosomes. These libraries are then used to sequence short fragments of both ends of the inserted sequence, which are mapped to the chloroplast genome to extract chloroplast reads. Many such pieces of chloroplast BAC inserts were used to ascertain which chloroplast structures exist in a given sample. BES reads can only provide information on chloroplast genome structure when a single read covers an entire IR region, with one end in LSC region and another end in the SSC region. Although a very useful method, BES reads may be limited for detecting highly atypical chloroplast genome structures, because only a short fragment from each end of a read is actually sequenced. For the restriction digestion method, one uses restriction enzymes to digest the chloroplast genome, and then decodes the chloroplast genome structure by studying the distribution of the resulting fragment lengths using agarose gel electrophoresis. Using this approach, Stein et al. (1986) found that the IR region was present in four different fragments which could be separated into two groups for which the sum of lengths was equal. They therefore concluded that chloroplast genome contained two equimolar isomers, which differed in their single copy orientation. The restriction digest method has provided much of the existing information on chloroplast genome heteroplasmy, but it is limited by the availability of suitable restriction sites (which may be unknown in some species), and requires a labor-intensive hybridization step to infer chloroplast genome heteroplasmy. Other methods have also been proposed, such as the use of PCR to attempt to amplify diagnostic regions of the chloroplast genome. However, it is not easy to generate PCR fragments which are longer than the IR region (usually 10–30 kb), so while PCR-based methods could only work well with short IRs (e.g., of a few hundred bp) (Wu, Lin, et al. 2011), their general application is likely to remain limited. Furthermore, PCR-mediated recombination could result in false positives when using PCR-based methods (Cronn et al. 2002; Lahr and Katz 2009). In short, current methods for detecting structural heteroplasmy in chloroplast genomes are labor- and time-intensive, which makes them difficult to employ for broad-scale studies. Current methods have been applied in a relatively small number of land plant species (*Beta vulgaris*, Brears et al. 1986; *Musa acuminata*, Martin et al. 2013; *Phaseolus vulgaris*, Palmer 1983; *Osmunda cIaytoniana*, *Osmunda cinnamomea*, and *Osmunda regalis*, Stein et al. 1986; and some species in the Pinaceae, Wu, Lin, et al. 2011), and as a result our understanding of the patterns and causes of structural heteroplasmy in chloroplast genomes remains somewhat limited.

In this study, we develop and apply a fast, simple, and cheap method to detect and quantify structural heteroplasmy in chloroplast genomes using long-read sequencing. Previous studies have used long-reads to detect recombination in organellar genomes (Shearman et al. 2016; Ruhlman et al. 2017; Dong et al. 2018; Wang, Song, et al. 2018). This study builds on those approaches to develop a targeted approach to detect and quantify chloroplast structural heteroplasmy using long-reads. This method requires just a single DNA extraction and sequencing run, followed by a simple bioinformatic analysis for which we provide free and open-source code (https://github.com/asdcid/Cp-hap). Our approach, which we call Cp-hap, relies on sequencing individual DNA molecules longer than the length of the IR region (roughly 10–30 kb in most species). Sequencing reads of this length are commonly obtained in great abundance when sequencing whole-plant DNA extractions on the Oxford Nanopore MinION device (Belser et al. 2018; Deschamps et al. 2018). Our method sifts through these long sequencing reads to find those that uniquely match 1 of the 32 uniquely identifiable structural haplotypes (supplementary fig. S1, Supplementary Material online), and then uses the abundance of these reads to estimate the relative abundance of each haplotype (details see Materials and Methods). Since many new plant samples are being sequenced with long-read sequencing technology, this method has the potential to provide many insights in the coming years, simply as a by-product of existing sequencing efforts.

We use Cp-hap to quantify the abundance of chloroplast genome structural haplotypes in 61 plant species. Our results suggest that most land plants contain the two commonly found chloroplast genome haplotypes in equal abundance whenever the chloroplast genomes contain a pair of large IRs. However, when the IR is small (e.g., only a few hundred bp in size) or repeat regions are not inverted but positioned, we find only a single haplotype. Finally, we show that multiple samples taken from a single individual all contain the same two haplotypes in equal abundance, suggesting that the process which maintains equimolar concentrations of the two haplotypes operates relatively rapidly, and consistent with the suggestion that flip-flop recombination is the underlying cause.

## Materials and Methods

### Cp-Hap, a New Method for Quantifying Chloroplast Structural Haplotypes Using Long-Read Sequencing Data

New long-read sequencing methods such as those developed by Oxford Nanopore can routinely sequence single DNA molecules of 10 s of kb in length. These molecules can be used to provide direct evidence for the existence and relative abundance of a large range of chloroplast genome haplotypes. Furthermore, long-reads from the chloroplast genome are often highly abundant in plant genome sequencing projects, because the chloroplast genome is typically present in copy numbers at least two orders of magnitude higher than the nuclear genome (Daniell et al. 2016; Morley and Nielsen 2016). Thus, most whole genome sequencing projects of plant provide abundant information for quantifying chloroplast structural haplotypes. Here, we describe a simple approach that uses this long-read sequencing data to estimate and quantify chloroplast structural haplotypes. We call this approach the Cp-hap pipeline, and provide open source code and detailed instructions for running this pipeline on GitHub at https://github.com/asdcid/Cp-hap.

The Cp-hap pipeline consists of three steps: 1) create a fasta file that contains all haplotypes of interest; 2) map the long-reads to the potential structures in step (1); and 3) use the results of step (2) to quantify the relative abundance of all possible haplotypes. Below, we describe each of these steps in more detail.

Step (1) requires us to create a fasta file containing all of the structural haplotypes of interest. We create this file based on a user-provided input file that contains the sequences of each of the LSC, SSC, and IR regions. To create a list of all possible haplotypes from the LSC, SSC, and IR regions, we first consider that each of these regions can have four different orientations: original, reversed, complement, and reverse-complement (supplementary fig. S2*A*, Supplementary Material online). This results in 256 possible structural haplotypes (256 = 4×4×4×4) in which the regions retain the ordering conserved across plant chloroplast genomes of LSC–IRA–SSC–IRB. These 256 haplotypes can be grouped into 128 identifiable structures, since it is not possible to distinguish between one structure and its direct complement given the two-stranded nature of the DNA molecule.

To uniquely identify 1 of these 128 structures, a single sequencing read would need to cover at least some parts of all four regions (LSC, SSC, and the two IR regions), for which the read would need to be at least 30–50 kb. This is because to cover all four regions, at a minimum a read must entirely cover the SSC (∼20 kb) region and one IR region (10–30 kb) and at least partially cover the LSC region and the other IR region. But the abundance of reads of this length tends to be relatively low in most data sets, meaning that in most cases it is almost impossible to uniquely identify 1 of the 128 possible structures by simply mapping a read to each structure.

To reduce the length of long-reads required to uniquely identify chloroplast structural haplotypes, the Cp-hap pipeline assumes by default that the two large repeat regions are always inverted. When assuming that the IR regions are always inverted, there are only 32 uniquely identifiable chloroplast genome structural haplotypes (supplementary fig. S1, Supplementary Material online). In this situation, a read only needs to entirely cover one IR region and partially cover the two adjacent LSC and SSC regions to provide evidence to uniquely identify 1 of the 32 structures (supplementary fig. S2*B*, Supplementary Material online). Because of this, this approach can provide direct evidence for 1 of the 32 possible structures using reads that are just 10–30 kb in size (i.e., at least as long as the length of a single IR region). Reads of this length are often highly abundant in long-read data sets, meaning that the default Cp-hap method is readily applicable to many long-read data sets being produced today.

In step (1), the default Cp-hap pipeline creates a fasta file of the 32 possible structural haplotypes when assuming that the IRs are inverted by simply combining the LSC, SSC, and IR region sequences with different orientations. After creating each structural haplotype, we then duplicate and concatenate it, such that single reads which span the point at which the genome was linearized will still align successfully to the relevant haplotype in the fasta file. This creates a fasta file with 32 sequences, each of which represents 1 of the 32 uniquely identifiable chloroplast structural haplotypes, and each of which contains a sequence that is twice as long as the chloroplast genome of interest.

In step (2) of the pipeline, we align our long-reads to our reference set of 32 possible structural haplotypes with Minimap2 (Li 2016), using “–secondary=no” setting to ensure that only the best alignment for each read is retained. After aligning all long-reads to the set of 32 possible structural haplotypes, we examine each mapped read, and retain only those that completely cover a whole IR region and at least 1 kb of the adjacent LSC and SSC regions. We then consider that these reads, which we term valid reads, uniquely identify 1 of the 32 possible structural haplotypes. Because our approach relies on correctly identifying reads from the chloroplast genome, it may be misled if the input data contain sequencing reads from other plastid genomes (such as chromoplast or leucoplast) that have nearly identical sequences (e.g., >90%) to those of the chloroplast genome.

In step (3) of the Cp-hap pipeline, we calculate the relative proportion of each of the 32 possible haplotypes. To do this, we simply divide the number of valid reads that aligned to each of the haplotypes by total number of the valid reads. These proportions provide direct estimates of the relative abundance of all 32 uniquely identifiable chloroplast genome structural haplotypes.

However, we acknowledge that the IR regions are not always the case. For example, the large repeat regions are positioned in-line instead of inverted in *Selaginella tamariscina* chloroplast genome (Xu et al. 2018). In cases such as this, Cp-hap pipeline is also able to figure out the structure. Here, we provide an example that how Cp-hap pipeline confirms a chloroplast genome with atypical structure, such as a paired of in-line repeats (supplementary fig. S2*C*, Supplementary Material online). Since Cp-hap duplicates and concatenates all default 32 structures (with IRs), reads from chloroplast genome with in-line repeats are able to map to Block A and/or C region of LSC_IR_SSC_IRrc structure (1 of the 32 default structure in Cp-hap pipeline), or Block B region of LSC_IRrc_SSC_IR structure (the other default structure in Cp-hap pipeline). Those reads are impossible to map to Block B region of LSC_IR_SSC_IRrc, or Block A or C region of LSC_IRrc_SSC_IR. Therefore, if some reads only map to Block A and/or C region of LSC_IR_SSC_IRrc and Block B region of LSC_IRrc_SSC_IR, it suggests that the chloroplast genome acquires in-line repeats.

In sum, Cp-hap pipeline can provide direct evidence to identify 1 of the 32 chloroplast structural haplotypes with a pair of IRs, and provide indirect evidence to identify other 96 chloroplast genome structural haplotypes with a pair of noninverted repeats.

### Assessing Chloroplast Structural Heteroplasmy across the Plant Tree of Life

We used the Cp-hap pipeline to assess chloroplast genome structural heteroplasmy across the plant tree of life. To do this, we searched for publicly available long-read data from land plants in the National Center for Biotechnology Information (NCBI) Sequence Read Archive (SRA) database (https://www.ncbi.nlm.nih.gov). We only selected data sets in which the average read length was >9 kb and for which the total amount of sequence data was >5 Gb. About 90 of the 164 potential species with data sets met this requirement in April, 2019.

For the 90 species, 19 of them do not have available chloroplast reference genomes. Due to the relative conservation of land plant chloroplast genomes, we used the chloroplast genome in the same genus as the reference genome for 17 species (supplementary table S1, Supplementary Material online). For the final two species, *Herrania umbratica* and *Siraitia grosvenorii*, we assembled and annotated chloroplast genomes de novo using long and short-reads (supplementary table S1, Supplementary Material online) following the pipeline described in (Wang, Schalamun, et al. 2018). The chloroplast genomes of *Herrania umbratica* and *Siraitia grosvenorii* have been deposited in NCBI (MN163033 and MK279915).

Full details of the reads, accession numbers, and chloroplast genomes used for each of the 90 species in our data set are given in supplementary table S1, Supplementary Material online.

### Chloroplast Structural Haplotype Quantification

For the 90 species for which we have long-read data, we ran the Cp-hap pipeline using default settings to generate the 32 possible haplotypes from the LSC, SSC, and IR regions. We then ran the rest of the Cp-hap pipeline with default settings for all species, resulting in measurements for each species of the number of valid reads that mapped to each haplotype, and the relative abundance of each haplotype. We calculated the relative abundance of each haplotype only if the species had more than five valid reads in total. This filter led to us excluding 29 data sets that did not have more than five valid chloroplast reads, resulting in a final data set of 61 species for which we have sufficient long-read data to estimate relative haplotype abundance.

For the *Eucalyptus pauciflora* data set, we quantified the chloroplast structural haplotypes not only for a single individual but also for eight separate samples taken from different branches of the same individual. This was possible because this data were sequenced from leaves collected from eight different branch tips from a single individual plant, and each sample contained sufficient long chloroplast reads to provide reliable haplotype quantification. This provides a unique opportunity to understand whether the relative abundance of chloroplast structural haplotypes varies or remains constant within an individual, potentially providing insights into the mechanisms which lead to the existence of multiple haplotypes.

### Statistical Analyses

**Fig. 1.**
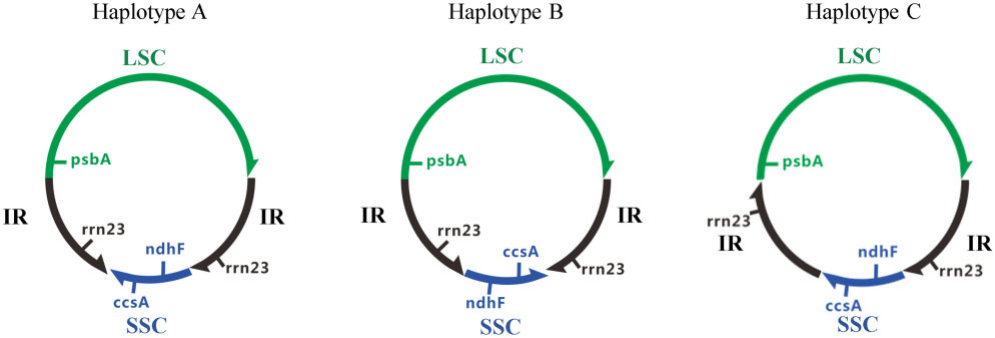
—The three different structural haplotypes of chloroplast genomes detected in this study. The green region is the long single copy (LSC) region and blue region is the short single copy (SSC) region. The two black regions are the Inverted Repeat (IR) regions. The arrow denotes 5′–3′ orientation. *psbA* is in the minus strand of LSC region, whereas *rrn23* is in the plus strand of IR regions. *ndhF* is in the minus strand of SSC region, while *ccsA* is in the plus strand of SSC region. For ease of communication, we use the relative order of three genes (*psbA* in LSC, and *ndhF* and *ccsA* in the SSC) to label these two haplotypes “A” and “B.” In haplotype A, these genes are ordered *psbA–ndhF–ccsA*. In haplotype B these genes are ordered *psbA–ccsA–ndhF*. For haplotype C, these three genes are ordered the same as haplotype A, but the repeat regions are in-line rather than inverted.

Although our method can detect a large number of structural haplotypes, across all of the species, we examined with inverted repeats, we observed three haplotypes, haplotypes A, B, and C (fig. 1).

Because no species contained more than two haplotypes, we used a binomial test to ask whether the abundances of haplotype A and B differ from a 1:1 ratio in each species, or in each individual sample in the case of *Eucalyptus pauciflora*. A significant result from a binomial test suggests that the observed haplotype abundances are unlikely to be explained by an underlying 1:1 ratio of chloroplast structural haplotypes.

In addition, since we have data for eight different branch tips of a single *Eucalyptus pauciflora* individual, we used two additional approaches to test for differences between samples. First, we used a χ^2^ test to ask whether the abundances of the two haplotypes differ among samples. Second, we tested for phylogenetic signal among the relative abundance of haplotypes observed in the eight branch tips. To do this, we used the structure of the physical tree (with branch lengths in units of centimeters) and the phylosignal package (Keck et al. 2016) in R (R Core Team 2013). This package implements five commonly used tests of phylogenetic signal: 1) Abouheif’s Cmean (Abouheif 1999); 2 and 3) Blomberg’s *K* and K* (Blomberg et al. 2003); 4) Moran’s I (Moran 1950); and 5) Pagel’s Lambda (Pagel 1999). These tests use different approaches to ask whether the physical structure of the individual tree helps to explain the observed variation in the relative haplotype abundance of the eight samples. For example, if the process that generates different haplotypes operates relatively slowly with respect to the age of this individual, which is roughly 40 years, then we might expect haplotype frequencies to vary due to genetic drift, and thus for the haplotype frequencies in neighboring parts of the tree to be more similar than would be expected by chance. This similarity would be revealed by the detection of significant phylogenetic signal in the relative abundance data. However, if the process that generates the different haplotypes operates very quickly with respect to the age of this individual, then we would expect all haplotype frequencies to be roughly equal, and thus, we would expect to observe no phylogenetic signal in the haplotype abundance data.

## Results

### Data Collection

We searched the NCBI SRA database, and selected 90 land plants species which met our length and data amount requirement (see Materials and Methods) to analysis (supplementary table S1, Supplementary Material online). Two of these species (*Herrania umbratica* and *Siraitia grosvenorii*) lacked suitable chloroplast reference genomes, so we therefore assembled these two chloroplast genomes de novo (see Materials and Methods). The chloroplast genomes of *Herrania umbratica* and *Siraitia grosvenorii* were 158,475 and 158,757 bp in size, consisting of a LSC (88,369 and 87,625 bp), a SSC (19,040 and 18,556 bp), and two IRs (25,533 and 26,288 bp), and encoding 134 and 133 genes, respectively (supplementary fig. S3, Supplementary Material online).

### Chloroplast Structural Haplotypes across the Plant Tree of Life

**Fig. 2.**
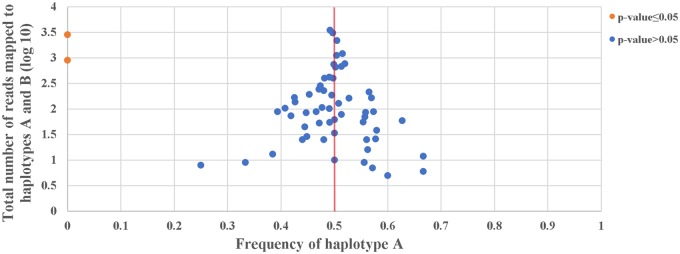
—The relationship between haplotype counts and haplotype frequencies. Each point is one species. The frequency of 0.5 is indicated by a red line. A blue dot means binomial *P* value >0.05, whereas the orange dot means binomial *P* value ≤0.05. Two species, *Pinus taeda*, *Picea sitchensis* only showed evidence of haplotype B, therefore the frequencies of them were 0. For the remaining 58 species, they show higher level of equal frequency between haplotype A and B with the increase of sample size. One species (*Selaginella tamariscina*) is omitted from this figure because it contained only a third haplotype (haplotype C, fig. 1).

For these 90 species, we obtained adequate long-read sequencing data to assess chloroplast genome structural heteroplasmy for 61 land plants species. Across all 61 species, we find just three structural haplotypes (fig. 1 andtable 1). All 58 of the Angiosperms in our data set are heteroplasmic, and contain roughly equal frequencies of haplotypes A and B (binomial test for departure from equal frequency, *P* value >0.05 in all cases). The remaining three species are not heteroplasmic (binomial test for departure from equal frequency $P=2.2\times 10^{-16}$ in all cases). The two Gymnosperms in our data set both contain only haplotype B, and one of the Pteridophytes in our data set (*Selaginella tamariscina*) contains only haplotype C. Figure 2 shows clearly that, as expected, there is a trend for data sets with larger sample sizes to show relative haplotype abundances closer to exactly 50/50.

**Table 1:** The Frequency of Existing Chloroplast Genome Haplotypes from 61 Species

Division	Order	Species	Size (bp)	LSC (bp)	SSC (bp)	IR (bp)	HA	HB	Frequency	*P* value
Angiospermae	Poales	*Andropogon gerardii*	139,712	81,548	12,592	22,786	31	31	0.50	1.00
		*Brachypodium mexicanum* ^a^	∼135,199	∼79,447	∼12,668	∼21,542	403	373	0.52	0.30
		*Eleusine coracana* ^a^	∼135,151	∼80,667	∼12,646	∼20,919	14	11	0.56	0.69
		*Hordeum vulgare *	136,462	81,671	12,701	21,045	347	329	0.51	0.51
		*Oropetium thomaeum* ^a**^	∼133,880	∼79,181	∼12,591	∼21,054	27	28	0.49	1.00
		*Oryza coarctata*	134,750	80,816	12,334	20,800	40	38	0.51	0.91
		*Oryza longistaminata *	134,567	80,596	12,357	20,807	8	4	0.67	0.39
		*Oryza meyeriana*	136,133	81,832	12,499	20,901	5	5	0.50	1.00
		*Panicum miliaceum*	140,048	81,841	9,603	24,302	37	22	0.63	0.07
		*Saccharum officinarum *	141,176	83,046	12,540	22,795	51	56	0.48	0.70
		*Saccharum spontaneum*	141,168	83,046	12,544	22,789	25	28	0.47	0.78
		*Sorghum bicolor*	140,754	83,733	12,503	22,259	94	71	0.57	0.09
		*Zea mays*	140,384	82,352	12,536	22,748	564	555	0.50	0.30
		*Zoysia japonica*	135,854	81,348	12,582	20,962	35	54	0.39	0.06
	Zingiberales	*Musa schizocarpa* ^a^	∼169,503	∼87,828	∼11,487	∼35,094	192	210	0.48	0.40
	Asparagales	*Yucca aloifolia* ^a**^	∼157,785	∼86135	∼18,292	∼26,679	87	105	0.45	0.22
		*Yucca filamentosa *	157,785	86,135	18,292	26,679	200	202	0.50	0.96
	Ranunculales	*Papaver somniferum*	152,931	83,029	17,920	25,991	12	13	0.48	1.00
	Ericales	*Actinidia eriantha*	156,964	88,759	20,541	23,832	122	94	0.56	0.11
	Lamiales	*Osmanthus fragrans *	155,326	86,564	17,370	25,696	65	63	0.51	0.93
		*Salvia splendens* ^a**^	∼153,995	∼84,573	∼17,590	∼25,916	15	11	0.58	0.56
	Solanales	*Cuscuta campestris*	86,749	50,978	7,063	14,354	5	8	0.38	0.58
		*Nicotiana attenuata*	155,886	86,602	18,518	25,383	3	6	0.33	0.51
	Asterales	*Lactuca sativa*	152,765	84,103	18,596	25,033	39	31	0.56	0.40
	Cucurbitales	*Siraitia grosvenorii*	158,757	87,625	18,556	26,288	5	4	0.56	1.00
	Fabales	*Ammopiptanthus nanus*	154,084	84,070	18,014	26,000	31	43	0.42	0.24
		*Arachis hypogaea*	156,391	85,946	18,797	25,824	17	17	0.50	1.00
		*Vigna unguiculata*	152,415	81,822	17,425	26,584	4	3	0.43	1.00
	Malpighiales	*Populus trichocarpa*	157,033	85,129	16,600	27,652	58	78	0.39	0.40
		*Salix purpurea*	155,590	84,454	16,220	27,458	41	47	0.47	0.59
	Rosales	*Fragaria vesca*	155,691	85,606	18,173	25,956	31	25	0.55	0.51
		*Fragaria ananassa *	155,549	85,532	18,145	25,936	4	2	0.67	0.69
		*Prunus avium* ^a**^	∼157,859	∼85,977	∼19,120	∼26,381	114	128	0.47	0.40
		*Rosa chinensis*	156,546	85,727	18,754	26,030	5	4	0.56	1.00
		*Rubus occidentalis* ^a^	∼155,760	∼85,430	∼18,768	∼25,781	9	7	0.56	0.80
	Fagales	*Betula pendula*	161,147	89,428	19,607	26,056	22	16	0.58	0.42
	Brassicales	*Arabidopsis thaliana*	154,478	84,170	17,780	26,264	135	150	0.47	0.41
		*Brassica napus *	152,860	83,030	17,760	26,035	51	38	0.57	0.20
		*Brassica oleracea*	153,364	83,136	17,834	26,197	1,714	1,773	0.49	0.33
		*Brassica rapa*	153,483	83,282	17,775	26,213	1,097	1,077	0.51	0.68
	Malvales	*Bombax ceiba*	158,997	89,022	21,111	24,432	71	96	0.43	0.06
		*Gossypium arboreum*	160,230	88,722	20,274	25,617	38	47	0.45	0.39
		*Gossypium barbadense *	160,317	88,841	20,294	25,591	11	14	0.44	0.69
		*Gossypium bickii*	159,422	88,073	20,183	25,583	333	330	0.50	0.94
		*Gossypium darwinii*	160,378	88,906	20,266	25,603	93	95	0.49	0.94
		*Gossypium hirsutum*	160,301	88,817	20,280	25,602	623	586	0.52	0.30
		*Gossypium longicalyx*	160,241	88,667	20,278	25,648	205	213	0.49	0.73
		*Gossypium mustelinum *	160,313	88,824	20,271	25,609	50	52	0.49	0.92
		*Gossypium raimondii*	160,161	88,654	20,205	25,651	48	38	0.56	0.33
		*Gossypium tomentosum *	160,433	88,932	20,273	25,614	42	61	0.41	0.08
		*Theobroma cacao*	160,619	89,333	20,194	25,546	86	77	0.52	0.53
	Myrtales	*Eucalyptus albens* ^a**^	∼160,076	∼88,828	∼18,476	∼26,386	20	25	0.44	0.55
		*Eucalyptus marginata *	160,076	88,828	18,476	26,386	13	16	0.45	0.71
		*Eucalyptus melliodora*	160,386	89,073	18,557	26,378	371	373	0.50	0.97
		*Eucalyptus pauciflora*	159,942	88,787	18,421	26,367	1,431	1,459	0.50	0.62
		Branch tip A					101	110	0.48	0.63
		Branch tip B					186	184	0.50	0.96
		Branch tip C					203	191	0.51	0.58
		Branch tip D					132	114	0.54	0.28
		Branch tip E					186	189	0.50	0.92
		Branch tip F					234	255	0.48	0.37
		Branch tip G					141	150	0.48	0.64
		Branch tip H					248	266	0.48	0.45
	Sapindales	*Acer yangbiense* ^a**^	∼156,262	∼86,018	∼18,072	∼26,086	104	94	0.53	0.52
		*Citrus maxima*	160,133	87,739	18,395	26,999	2	6	0.25	0.29
		*Xanthoceras sorbifolium*	161,231	85,299	18,692	28,620	110	119	0.48	0.60
Gymnosperm	Pinales	*Pinus taeda*	121,530	66,444	54,288	399	0	898	0.00	2.2e-16^b^
		*Picea sitchensis*	124,049	67,411	55,758	440	0	2,845	0.00	2.2e-16^b^
Pteridophytes	Selaginellales	*Selaginella tamariscina* ^c^	126,399	53,176	47,573	12,825	0	0	—	—

a The long-reads were mapped to the chloroplast genome from the same genius species due to the lack of chloroplast genome of this species, so the lengths of LSC, SSC, and IR are around (∼).

b *P* value ≤0.05.

c *Selaginella tamariscina* only present one haplotype, haplotype C, with 174 counts, and *P* value is 2.2e-16.

Size, the chloroplast genome size; HA/B, haplotype A/B, the number of supported reads of LSC and SSC having identical/opposite orientation; frequency, the proportion of the count of haplotype A in the count of haplotype A + B.

Across eight different samples taken from a single large *Eucaltyptus pauciflora* plant (table 1), we detected roughly equal frequencies of haplotypes A and B in all eight samples (binomial *P* values >0.05 in all cases), no evidence for differences in relative frequencies among samples (χ^2^*P* value >0.05), and no evidence for phylogenetic signal in the any of the relative abundances (*P* value >0.05 in all tests), where the underlying topology and branch lengths are taken to be the physical structure of the tree itself (see Materials and Methods).

## Discussion

In this study, we develop a new method to detect and quantify structural heteroplasmies in the chloroplast genome. By applying this method to a broad range of plant species including representatives from Angiosperms, Gymnosperms, and Pteridophytes, we show that of the large number of possible structural haplotypes, only a very small number are observed in nature. For example, species with IRs almost universally contain the same two structural haplotypes in ratios that do not depart significantly from 50/50. The only exceptions to this are species in which the repeats are not inverted (e.g., one of the Pteridophytes in our data set), or in which the repeats are inverted but much reduced in length (e.g., both of the Gymnosperms in our data set). In these species, we detect just one haplotype. Our results demonstrate not only that the relative abundance of the two most common haplotypes stable across the Angiosperm tree of life (i.e., all 58 Angiosperms in our data set had a frequency that was not different from 50/50) but that it also stable within an individual plant (i.e., within a single large plant, all sampled leaves contained both haplotypes at a ratio that was not different from 50/50). This suggests that the process which maintains the equal frequencies of the two commonly observed haplotypes operates relatively rapidly.

The most parsimonious interpretation of our results suggests that structural heteroplamsy should be extremely common across all angiosperms. In light of this, it may be necessary to re-examine many previous suggestions of structural differences in the chloroplast genomes of angiosperms *between* species (Ibrahim et al. 2006; Yang et al. 2010; Liu et al. 2013; Zhang et al. 2014; Wang et al. 2015), as Walker et al. (2015) suggested. For example, Ibrahim et al. (2006) implied that the SSC of *Gossypium barbadense* was inverted when compared with *Gossypium hirsutum*. However, our study shows that both *Gossypium barbadense* and *Gossypium hirsutum* contain two structural haplotypes (haplotypes A and B; table 1), thus showing that there are no structural differences between these two species. Future studies may benefit from assuming at the outset that it is highly likely that the chloroplast genome of any angiosperm species, particularly if it contains two long inverted repeats, is most likely to exist in two roughly equimolar haplotypes, haplotypes A and B shown in figure 1. Although the Gymnosperms and Pteridophytes in this study lacked long inverted repeats and had just one structural haplotype, it is plausible that if Gymnosperms and Pteridophytes species are found with chloroplast genomes that contain two long inverted repeats, they may also contain two structural haplotypes.

Of the 61 species for which we had sufficient data, we find just three species in which there is no evidence for structural heteroplasmy in the chloroplast genome: *Selaginella tamariscina*, *Pinus taeda*, and *Picea sitchensis*. *Selaginella tamariscina* is a pteridophyte in which the repeats in the chloroplast genome are positioned in-line rather than inverted (Xu et al. 2018). The existence of a single structural haplotype in this species is therefore consistent with the hypothesis that the process which generates chloroplast structural heteroplasmy is mediated by long IRs. In the gymnosperms *Pinus taeda* and *Picea sitchensis*, the repeats are inverted but their lengths are highly reduced from the typical 10–30 kb in most species to just 399 and 440 bp, respectively. The existence of a single structural haplotype in these species is also consistent with the flip-flop recombination theory, because it is feasible that the highly reduced IRs preclude the formation of the dumbbell-like structure (Stein et al. 1986) which is necessary to activate flip-flop recombination. Whether or not flip-flop recombination is the underlying cause, our results confirm that chloroplast structural heteroplasmy appears to require the existence of two long IRs in the chloroplast genome, and therefore that groups which lack this trait would be expected to lack chloroplast structural heteroplasmy. For example, chloroplast genomes in the Pinaceae and Cupressophytes (two major groups of gymnosperms) usually lack one IR or have IRs that are highly reduced in size (Raubeson and Jansen 1992; Wu, Lin, et al. 2011; Wu, Wang, et al. 2011; Yi et al. 2013; Guo et al. 2014; Wu and Chaw 2016; Qu et al. 2017), and we therefore expect that these species would lack chloroplast structural heteroplasmy.

In this study, we focused on examining heteroplasmies in the quadripartite structure of the chloroplast genome. However, other smaller structural heteroplasmies have been observed in some chloroplast genomes, and their existence challenges the conclusion that long inverted repeats are a prerequisite for the existence of structural heteroplasmy in the more general sense. Although flip-flop recombination may be absent when the IR regions are small (see above), some studies have suggested that homologous recombination could induce structural heterplasmy from other much smaller inverted repeats in chloroplast genomes (Guo et al. 2014; Qu et al. 2017). For instance, the *trnQ-UUG* gene (∼150 bp) is duplicated in the LSC region in some *Juniperus* species, and this duplication is associated with a 36 kb inversion resulting in the coexistence of two isomeric chloroplast genome structures, although their abundance is unequal (0.8–5.0%) (Guo et al. 2014). Qu et al. (2017) reported a similar finding in other Cupressoideae species, and suggest that even a pair of very small inverted repeats (11 bp) could induce a 34 kb inversion and resulting structural heteroplasmy in *Calocedrus macrolepis*. In light of these results, it remains unclear why the highly reduced IRs of *Pinus taeda* and *Picea sitchensis* studied here fail to induce structural heteroplasmy, while much shorter inverted repeats in regions of the chloroplast genome outside the IR region apparently do induce structural heteroplasmy. One possibility is that different processes mediate the formation of structural heteroplasmies in these cases (Guo et al. 2014; Qu et al. 2017). Another possibility is that the frequency of the alternative haplotype is too low for us to detect in *Pinus taeda* and *Picea sitchensis*. However, if this were the case, then the frequency of haplotype A in these species would have to be extremely low: for example, in *Picea sitchensis*, we detected 2845 long-reads supporting haplotype B and no reads supporting haplotype A.

We note that there is evidence that not all chloroplast genomes are circular. For example, Bendich and Smith (1990) found the four different forms of chloroplast genome in watermelon, and Oldenburg and Bendich (2004) further stated that only 3–4% of maize chloroplast genomes are circular and later Oldenburg and Bendich (2016) sequenced the end of linear chloroplast genomes to precisely identify their four endpoints. The methods we present here would also detect structural heteroplasmies in monomer linear chloroplast genomes. Furthermore, it may be possible to extend the approaches we present here, particularly if combined with longer read lengths, to shed more light on the debate around the linearity or circularity of chloroplast genomes.

The Cp-hap pipeline we present here provides a convenient method for quantifying structural heteroplasmy in chloroplast genomes. It adds to suite of existing methods such as restriction digests (Palmer 1983; Stein et al. 1986) and BES (Martin et al. 2013), but differs from these methods in that it uses long sequencing reads to provide direct evidence for uniquely identifiable chloroplast structures. It is of note that the two available long-read sequencing methods, PacBio and Oxford Nanopore sequencing, differ in their utility for detecting chloroplast structural heteroplasmy. For example, only ∼63% (46 out of 73 species) of the PacBio data sets we used in this study contained enough long chloroplast reads to adequately measure chloroplast structural heteroplasmy. In contrast, ∼88% (15 out of 17 species) of Oxford Nanopore data sets provided enough long-read to measure the chloroplast genome heteroplasmy. Given the increasingly popularity of Oxford Nanopore sequencing, and ongoing improvements in the read-lengths available from PacBio sequencers, we hope that the simplicity of the Cp-hap pipeline will accelerate further work on chloroplast structural heteroplasmy. 

## Supplementary Material

Supplementary data are available at *Genome Biology and Evolution* online.

## Supplementary Material

evz256_Supplementary_Data

## Notes

**Data deposition:** The Herrania umbratica and Siraitia grosvenorii chloroplast genomes in this project have been deposited at NCBI under the accession MN163033 and MK279915.
