# Supplementary material for: Long-Reads Reveal That the Chloroplast Genome Exists in Two Distinct Versions in Most Plants
Source: Genome Biol Evol. 2019 Nov 21;11(12):3372–81. doi: 10.1093/gbe/evz256 (PMC7145664; doi:10.1093/gbe/evz256)

**FIG. S1.** The 32 chloroplast structural haplotypes in Cp-hap pipeline.


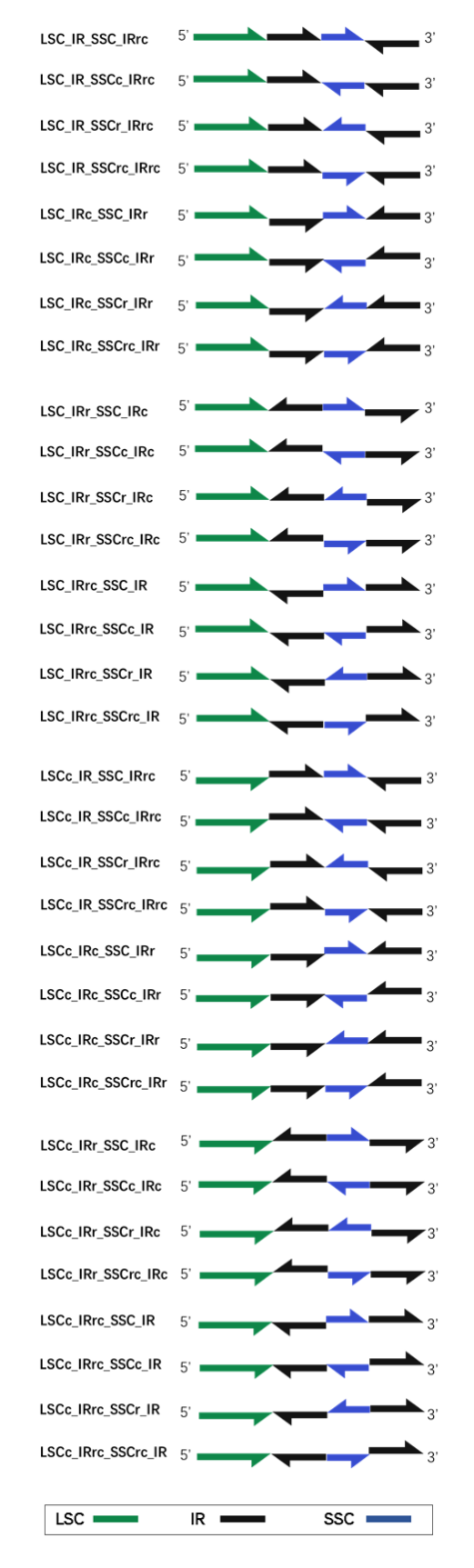


**FIG. S2.** The Cp-hap pipeline. (A) Each of the four regions can exist in four possible orientations: original, reversed, complement, reversed-complement. (B) An example that how to use long-reads to confirm chloroplast genome structure with a pair of IRs. Read A entirely covers an IR region and partially covers the LSC and SSC region, and can therefore only map to one haplotype. Read B entirely covers the SSC region and partially covers two IR regions, but it can map to both haplotypes. (C) An example of how to use long-reads to confirm chloroplast genome structure with a pair of non-inverted repeats. In this case the chloroplast genome is with in-line repeats. Reads located in the first repeat region, such as Read C, are only able to map to Block A and/or C region in LSC_IR_SSC_IRrc structure, but no read cannot map to Block B region in LSC_IR_SSC_IRrc structure. Reads positioned in the second repeat region, such as Read D, can only map to Block B region in LSC_IRrc_SSC_IR structure, but no read map to Block A or C region in LSC_IRrc_SSC_IR structure.


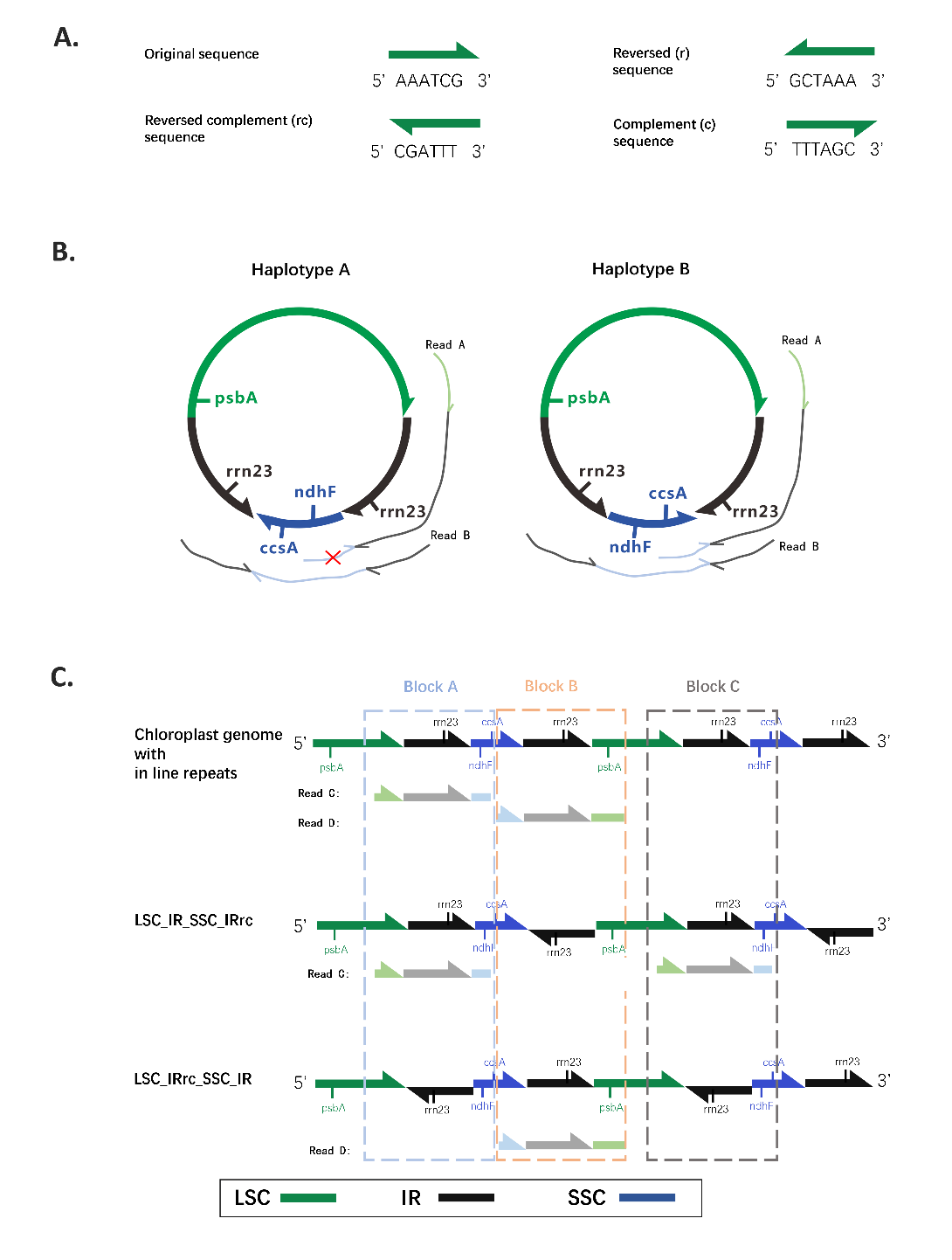


**FIG. S3.** The chloroplast genome annotation and structure of *Herrania umbratica* and *Siraitia grosvenorii*. Genes shown on the inside of the circle are transcribed clockwise, whereas genes shown on the outside of the circle are transcribed counter clockwise. The grey region in the inside circle shows the GC content across the chloroplast genome.

.
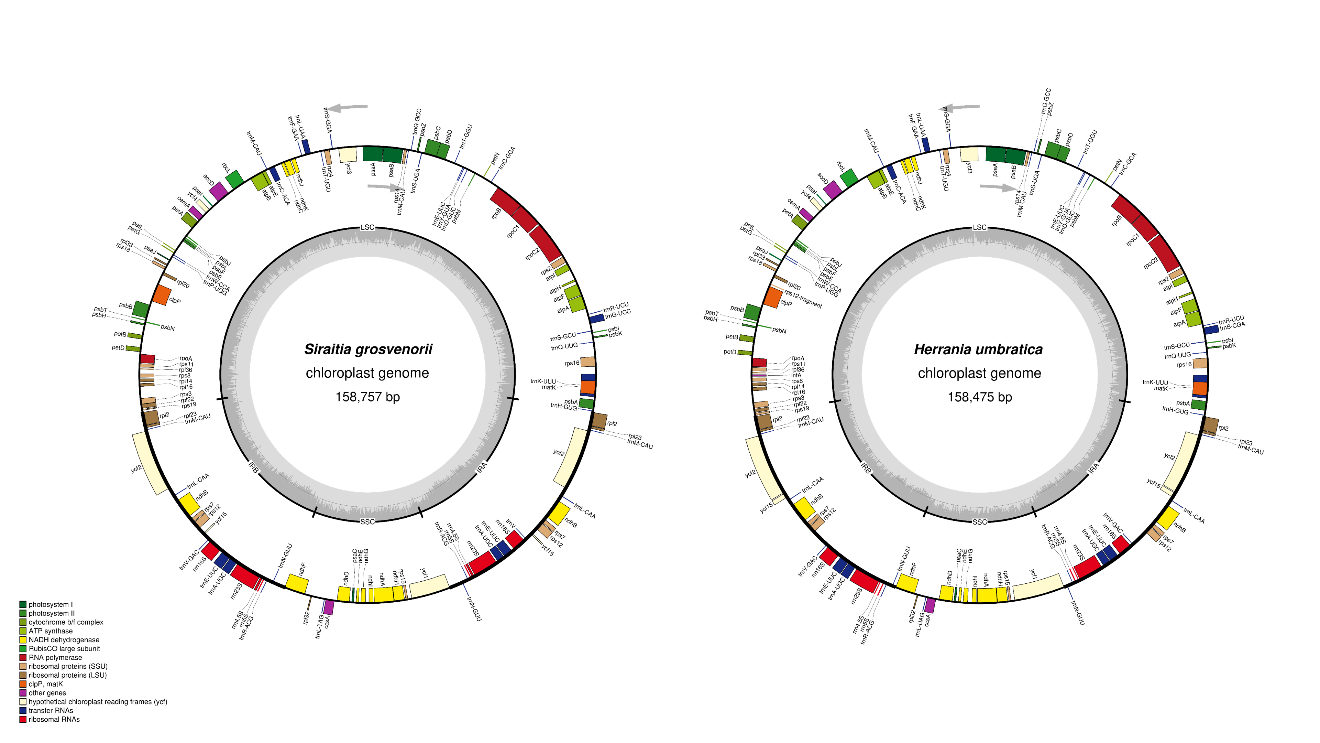

Supplement: evz256_Supplementary_Data [file evz256_supplementary_data.zip › supplymentary_fig_caption.docx]
