# Supplementary figures and images for: Long-Reads Reveal That the Chloroplast Genome Exists in Two Distinct Versions in Most Plants
Source: Genome Biol Evol. 2019 Nov 21;11(12):3372–81. doi: 10.1093/gbe/evz256 (PMC7145664; doi:10.1093/gbe/evz256)

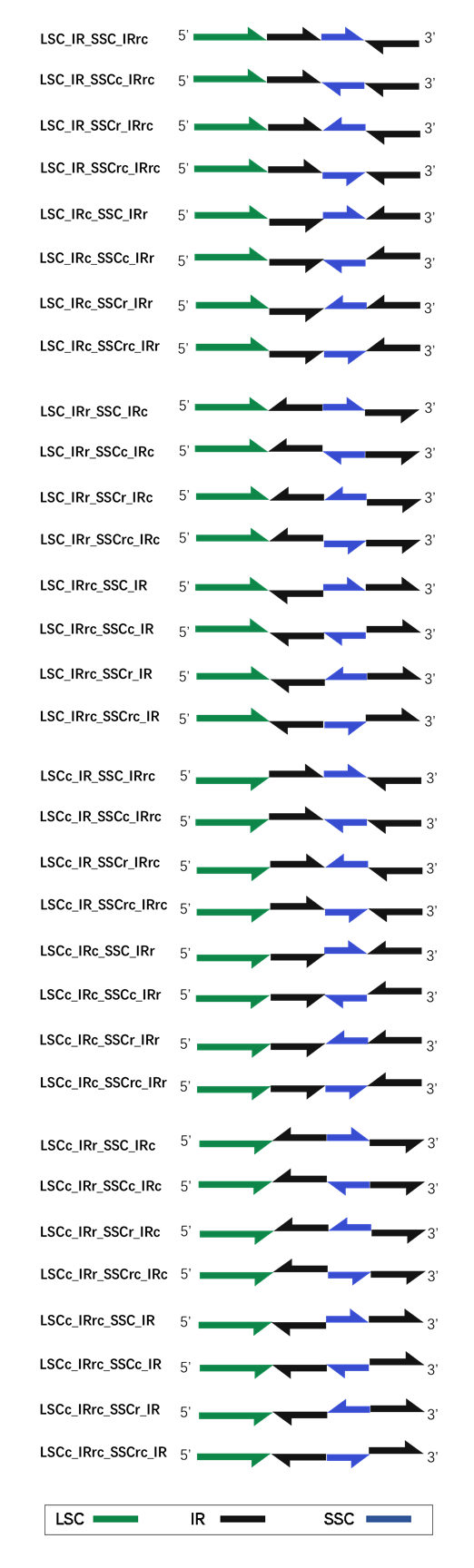

Supplement: evz256_Supplementary_Data [file evz256_supplementary_data.zip › Fig S1. 32 chloroplast genome sturctures.tif]

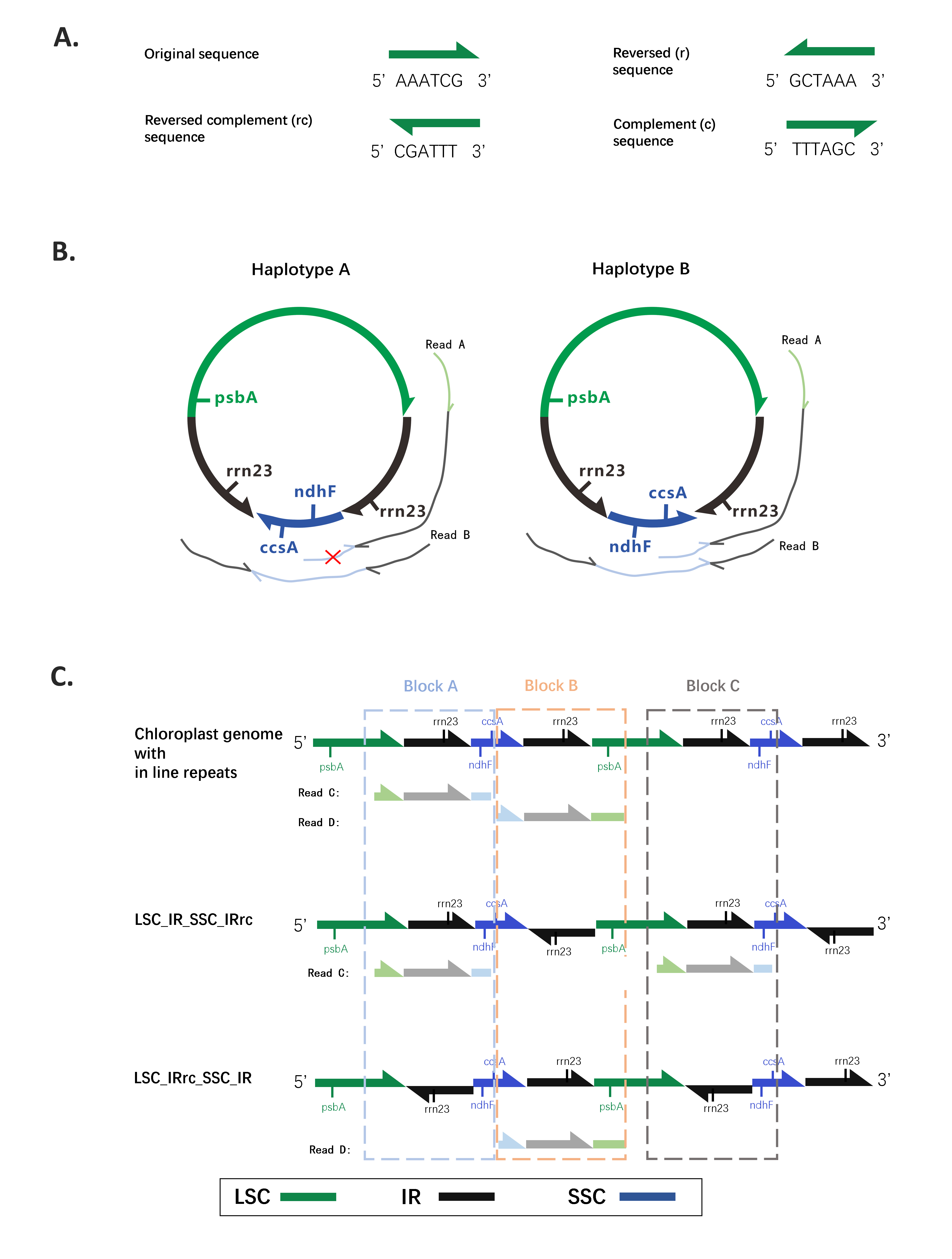

Supplement: evz256_Supplementary_Data [file evz256_supplementary_data.zip › Fig S2. Method.jpg]

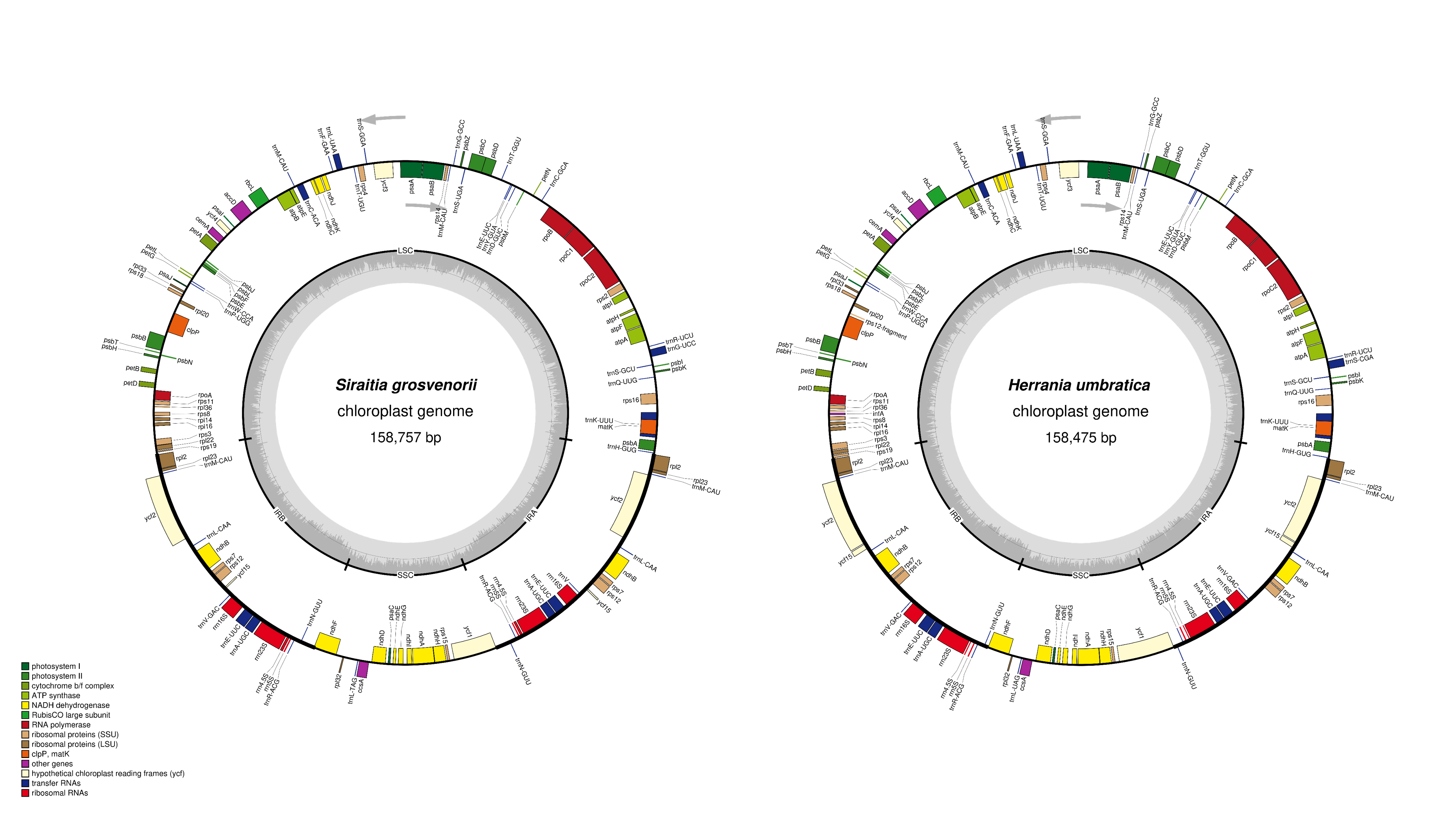

Supplement: evz256_Supplementary_Data [file evz256_supplementary_data.zip › Fig S3. Cp annotation.tif]
